# Isolation and Identification of the Anti-Oxidant Constituents from *Loropetalum chinense* (*R. Brown*) Oliv. Based on UHPLC–Q-TOF-MS/MS

**DOI:** 10.3390/molecules23071720

**Published:** 2018-07-14

**Authors:** Haifang Chen, Mulan Li, Chen Zhang, Wendi Du, Haihua Shao, Yulin Feng, Wugang Zhang, Shilin Yang

**Affiliations:** Jiangxi University of Traditional Chinese Medicine, Nanchang 330006, China; chenhf88@126.com (H.C.); 15570351171@163.com (M.L.); 18363259880@163.com (C.Z.); wendi_du_0216@163.com (W.D.); SHH_2013@tom.com (H.S.); slyang3636@126.com (S.Y.)

**Keywords:** *Loropetalum chinense* (*R. Brown*) Oliv., antioxidant constituents, isolation, identification, UHPLC-Q-TOF-MS/MS

## Abstract

The aim of this study was to identify the chemical constituents of *Loropetalum chinense *(*R. Brown*) Oliv. (LCO) and determine which of these had antioxidant effects. The chemical composition of a 70% ethanol extract of LCO was analyzed systematically using UHPLC–Q-TOF-MS/MS. The chemical components of the 70% ethanol extract of LCO were then separated and purified using macroporous resin and chromatographic techniques. Antioxidant activity was evaluated using a DPPH assay. In total, 100 compounds were identified tentatively, including 42 gallic acid tannins, 49 flavones, and 9 phenolic compounds. Of these, 7 gallium gallate, 4 flavonoid and 8 quinic acid compounds were separated and purified from the 70% ethanol extract of LCO. The compounds identified for the first time in LCO and in the genus Loropetalum were 3,4,5-trimethoxyphenyl-(6′-*O*-galloyl)-*O*-β-d-glucopyranoside, protocatechuic acid, ethyl gallate, 5-*O*-caffeoylquinic acid, 3-*O*-caffeoylquinic acid, 3,5-*O*-diocaffeoylquinic acid, 4,5-*O*-diocaffeoylquinic acid and 3,4-*O*-diocaffeoylquinic acid. The 50% inhibitory concentration (IC_50_) values of compounds 1,2,3,4,6-penta-*O*-galloyl-β-d-glucose, gallic acid, protocatechuic acid, and ethyl gallate were 1.88, 1.05, 1.18, and 1.05 μg/mL, respectively. Compared with the control group (V_C_) (2.08 μg/mL), these compounds exhibited stronger anti-oxidation activity. This study offered considerable insight into the chemical composition of LCO, with preliminary identification of the antioxidant ingredients.

## 1. Introduction

Reactive oxygen species (ROS) refers to oxygen-containing reactive species and includes superoxide anions (O_2_^−^), hydrogen peroxide (H_2_O_2_), and hydroxyl radicals (•OH) [1]. Under normal conditions, ROS are in a constant dynamic state of production and elimination in vivo. They play an important role in physiological metabolic processes such as enhancing leukocyte phagocytosis and prostaglandin synthesis, and participating in enzymatic pathways that contribute to immunity [2]. A net excess of ROS can follow an imbalance in ROS production and elimination. This can lead to a series of peroxidation reactions, cross-linking or breakages with subsequent cellular structural damage and dysfunction. If this occurs chronically, a number of pathophysiological processes may ensue including arteriosclerosis, cardiovascular diseases, neurodegenerative diseases, cancers, and other disorders associated with aging [3,4,5,6]. Therefore, elimination of excess ROS in the body is important for maintaining physiological health. Recently, there has been an increase in interest in antioxidants as substances that protect against oxidative damage. A particular focus has been secondary plant polyphenol or phenolic metabolites such as catechins, epigallocatechin gallate, and catechin-aldehyde polycondensates [7,8,9]. These phenolic compounds also possess other bioactivities including antimicrobial and anti-inflammatory effects [10,11]. Therefore, screening for antioxidants could be an effective strategy for sourcing new drugs or functional foods.

*Loropetalum*, as a member of the *Hamamelidaceae* family, contains three species (*Loropetalum lanceum* Hand-Mzt, *Loropetalum subcapitatum* Chun ex Chang, and *Loropetalum chinense* (*R. Brown*) Oliv.) as well as one variety (*Loropetalum chinense* var. rubrum) in China. Among them, *Loropetalum chinense* (*R. Brown*) Oliv. (LCO), an evergreen shrub or small arbor, was first recorded officially in the 1970 edition of the Chinese pharmacopoeia. LCO has antipyretic, hemostatic, and detoxificant effects, and is a traditional medicine widely used in the treatment of bleeding disorders, burns, skin infections, dysentery, and diarrhea [12]. Modern pharmacological research has shown that LCO has bacteriostatic, anti-inflammatory, healing, and antioxidant effects [13].

The multiple biological activities of LCO are attributed to its diverse constituents. Phytochemical studies reported the presence of tannins, flavonoids, lignans, terpenoids, and steroids in LCO [14,15,16,17]. Alkanes, aldehydes, and terpenoids have been identified as the dominant constituents of LCO essential oil [13]. However, there is little information on the biological constituents that have antioxidant effects. In earlier work, we used DPPH (2,2-diphenyl-1-picrylhydrazyl) radical scavenging activity to evaluate the antioxidant effects of four fractions (water, and 10%, 70%, and 95% ethanol eluates) separated with HPD-400 macroporous resin. Our results showed that the four fractions exhibited various antioxidant effects. The 50% inhibitory concentrations (IC_50_) were 22.53 μg/mL for the water eluate, 11.47 μg/mL for 10% ethanol eluate, 9.73 μg/mL for 70% ethanol eluate and 39.20 μg/mL for 95% ethanol eluate [18]. However, this work only assessed the antioxidant effects of various LCO fractions. Details of the specific constituents responsible for the antioxidant activities remain unclear.

Therefore, in the present study, we used UHPLC-Q-TOF-MS/MS to systemically analyze the chemical constituents of the ethanol extract of LCO to identify the components responsible for its antioxidant effects.

## 2. Materials and Methods

### 2.1. Chemicals and Reagents

DPPH and vitamin C (V_C_) were purchased from Sigma Chemicals (Shanghai, China). Chemical standards of gallic acid, protocatechuic acid, quercetin, kaempferol, and chlorogenic acid were obtained from Chengdu Munster Biotechnology Co., Ltd. (Chengdu, China). LC-MS grade methanol and HPLC grade methanol for use as solvents, and acetic acid, formic acid, and acetonitrile were purchased from Fisher Scientific (Shanghai, China). HPD-400 macroporous resin was purchased from Cangzhou Bon Adsorber Technology Co., Ltd (Jiangsu, China). Various types of silica gel were obtained from Qingdao Haiyang Chemical Co., Ltd (Shandong, China). Double distilled water was used in the LC mobile phase. All other chemicals used were analytical grade.

### 2.2. Plant Material and Extraction

LCO was collected in May around the city of Jindezhen (29°25′ N, 117°16′ E) (Jiangxi province, China). Plants were authenticated by Prof. Shi-lin Yang, Jiangxi University of Traditional Chinese Medicine. A voucher specimen (No. 20100521) was stored in the National Pharmaceutical Engineering Center for Solid Preparation in Chinese Materia Medica, Jiangxi University of Traditional Chinese Medicine.

Extraction parameters were as described in earlier work by our group [19]. Dried branches and leaves of LCO (17 kg) were milled and reflux-extracted twice with an 8-fold weight of 60% ethanol solution for 2 h. Extracts were then combined and filtered with vacuum suction filtration. The combined filtrates were concentrated to dryness under vacuum with a rotary evaporator to yield a yellowish-brown residue (2.754 kg) that was stored at 4 °C until needed. The residue was re-dissolved with methanol to give a final concentration of 10 mg/mL and then filtered through a 0.22 μm filter membrane before UHPLC analysis.

### 2.3. DPPH Assay

DPPH, control, blank and sample solutions were prepared as follows. DPPH solution: 3 mg DPPH was dissolved in absolute ethanol and mixed with 0.03 g/L reaction solution protected from light. Control: 1 mL solvent and 3 mL DPPH solution were combined in a test tube, shaken to mix and left to react for 30 min in the dark at room temperature (25 °C). Absorbance (A_0_) was then measured at a wavelength of 517 nm with a UV visible spectrophotometer (UV-2550, Shimadzu, Kyoto, Japan). Blank: Various concentrations of sample solution (1 mL) were added to 3 mL absolute ethanol in a test tube, shaken to mix, and then stored in the dark at 25 °C. After 30 min, absorbance (A_j_) was measured at 517 nm wavelength. Sample: Various concentrations of sample solution (1 mL) were mixed with 3 mL DPPH solution in a test tube, stored in the dark at 25 °C for 30 min before measuring the absorbance (A_i_) at a wavelength of 517 nm [20,21,22]. The scavenging rate was calculated as follows: scavenging activity (%) = [1 − (A_i_ − A_j_)/A_0_] × 100.

### 2.4. UHPLC–Q-TOF-MS/MS Analysis of the Crude Extract

Chromatographic analysis was carried out on a Prominence™ UHPLC system (Shimadzu, Japan) coupled with a triple TOF™ 5600+ MS/MS system (AB Sciex, Framingham, MA, USA). Separations were accomplished on a Welch C_18_ column (2.1 mm × 100 mm, 1.8 μm, Shanghai, China) and 2 μL injected into UHPLC. The column oven was maintained at 40 °C. A linear gradient elution of eluents A (water containing 0.1% formic acid) and B (acetonitrile) was used for separation. The elution program was: 0.1–2 min, 1–5% B; 2.01–20 min, 8–37% B; 20–22 min, 37–50% B; 22–32 min, 50–90% B; 32.01–35 min, 95–95% B, 35.01–40 min, 1% B. The flow rate was 0.25 mL/min.

The mass spectrometer was operated both in negative and positive ion modes. The following parameter settings were used: ion spray voltage 4500 V; turbo spray temperature 600 °C; curtain gas 25 psi, nebulizer gas (GS 1) 50 psi, heater gas (GS 2) 50 psi, declustering potential 100 V. TOF MS and TOF MS/MS were scanned with the mass range of *m*/*z* 50–1250 and 50–1250, respectively. In the IDA-MS/MS experiment, collision energy was set at 35 eV and collision energy spread was (±) 10 eV. Accurate mass and composition for the precursor and fragment ions were analyzed using Peakview software (Version 1.2, AB Sciex) integrated with the instrument.

### 2.5. Isolation of the Crude Extract

The crude extract solution was adsorbed by the HPD-400 macroporous resin and eluted with water, and 10%, 70%, and 95% ethanol-water. The 10% and 70% fractions were concentrated under vacuum to recover the organic solvent to dryness. They were then isolated using various chromatographic techniques including silica gel, Sephadex LH-20, ODS, and MCI columns, and re-crystallization methods.

The compounds were identified using UV, MS, ^1^H-NMR, and ^13^C-NMR experiments along with comparison of their spectroscopic properties from the literature. Their antioxidant activities were measured by DPPH assay.

## 3. Results

### 3.1. UHPLC–Q-TOF-MS/MS Analysis of the Crude Extract

**Figure 1:**
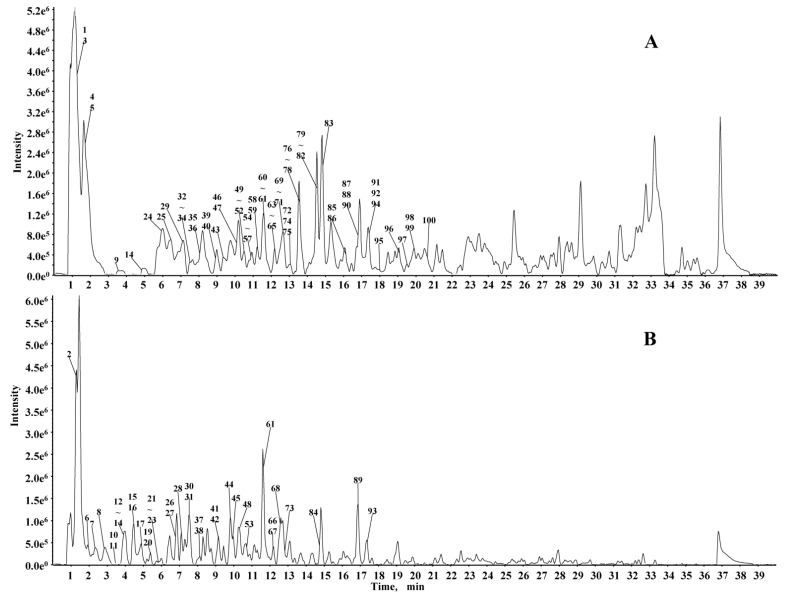
BPI chromatograms of *Loropetalum chinense *(*R. Brown*) Oliv. (LCO) in negative ion mode (**A**) and in positive ion mode (**B**).

To obtain the abundant constituents from LCO, UHPLC conditions (type of column, mobile phase system, column temperature, and flow rate) were first optimized followed by the MS conditions (capillary voltage, declustering potential, and collision energy). UHPLC-Q-TOF-MS/MS in positive (A) and negative (B) ion modes was employed to characterize the corresponding signals. The base peak chromatogram under optimized chromatographic and MS conditions are presented in Figure 1. Retention times, observed molecular weight, and fragment ions for each metabolite, and their identities are presented in Table 1. A total of 100 compounds were identified or tentatively characterized including 42 gallic acid tannins, 49 flavones, and 9 phenolic compounds. The structures of these compounds were tentatively assigned by matching the MS/MS data with a reference or public database such as PubChem (https://pubchem.ncbi.nlm.nih.gov/) or MassBank (http://www.massbank.jp/).

#### 3.1.1. Fragmentation of Gallic Acid Tannins

**Figure 2:**
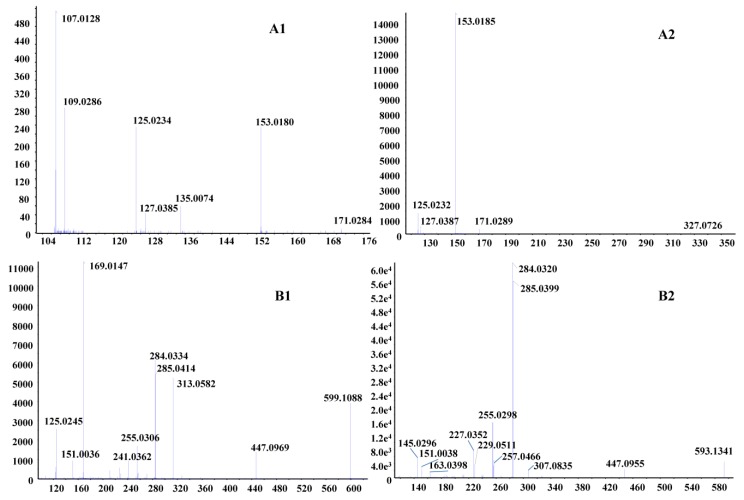
The MS/MS spectrum information of different compounds in LCO. (**A1**) Peak 5; (**A2**) Peak 4; (**B1**) Peak 66; (**B2**) Peak 89.

The 42 tannin compounds have gallic acid and glucose as their basic structural units, which contain or lose C_7_H_6_O_5_ (*m*/*z*: 170), -C_7_H_5_O_5_ (*m*/*z*: 169), -C_7_H_5_O_4_ (*m*/*z*: 153), -C_6_H_5_O_3_ (*m*/*z*: 125) and -C_6_H_11_O_5_ (*m*/*z*: 163) ion fragments in the MS/MS spectrum [23,24,25,26]. For example, in the positive ion of the MS/MS spectrum, the [M + H]^+^ ions of peak 5 were found at *m*/*z*: 171.0274, while *m*/*z*: 153.0180 (M + H-H_2_O), 135.0074 (M + H-2H_2_O), 125.0234 (M + H-HCHO_2_) and 107.0128 (M + H-HCHO_2_-H_2_O) fragment ions were consistent with gallic acid (C_7_H_6_O_5_). Therefore, peak 5 was determined to be gallic acid (Figure 2A1). Peak 54 had [M + H]^+^ ions at *m*/*z*: 303.0140, and *m*/*z*: 285.0035 (-C_14_H_5_O_7_), 275.0189 (-C_13_H_7_O_7_), 257.0082 (-C_13_H_7_O_6_) and 229.0130 (-C_12_H_5_O_5_) fragment ions were observed in the MS/MS spectrum, which were formed by loss of H_2_O, CO_2, _and CO. Following comparison with the reference substance, peak 54 was determined to be ellagic acid. Peak 4 had [M + H]^+^ ions at *m*/*z* 345.0818 in the MS/MS spectrum (Figure 2A2). Three fragment ion at *m*/*z*: 171.0289, 153.0185, and 125.0232 showed that peak 4 contained gallic acid units. Based on these MS/MS data, peak 4 was identified as theogallin (C_14_H_16_O_10_). Under the negative ion mode, peak 11 had [M − H]^−^ ions at *m*/*z* 331.0667, and fragment ions at *m*/*z* 169.0148, 151.0031, and 125.0257 indicating the presence of gallic acid units. [M − H] ions produce *m*/*z* 169.0148 by losing 162 Da (-C_6_H_10_O_5_), which indicated the presence of hexose units and thus peak 11 peak was determined to be monogalloylglucose.

#### 3.1.2. Fragmentation of Flavonoids Compounds

The Retro-Diels-Alder (RDA) cleavage reaction involves loss and rearrangement of flavonoid aglycone C rings in different ways. The main fragment was derived from cleavage of C-C and C-O bonds on the C ring, and neutral fragments such as CO, CO_2_, H_2_O, and C_2_H_2_O. Hexose (*m*/*z*: 162) and pentose (*m*/*z*: 132) fragments often appear in the MS/MS cracking spectrum of flavonoid glycosides [27,28,29]. In this study, flavonoid glycosides produced gallic acid (*m*/*z*: 169) fragments. For example, peak 71 was identified as quercetin (C_15_H_10_O_7_) through comparison with the reference substance. [M + H]^+^ ions at *m*/*z*: 303.0305, and RDA cleavage of C_1_-C_2_ and C_3_-C_4_ bonds in the C ring generated the fragment *m*/*z*: 153.0181. After breakage of the C_2_-O and C_10_-O bonds of the C ring, the B ring produced *m*/*z*: 137.0229 by losing two CO. [M + H]^+^ ion lost one H_2_O (18 Da) to form *m*/*z*: 285.0037, and one CO (28 Da) to form *m*/*z*: 275.0176 (-C_14_H_10_O_6_). Subsequently, *m*/*z*: 275.0176 (-C_14_H_10_O_6_) was produced *m*/*z*: 257.0436 (C_14_H_8_O_5_) and *m*/*z*: 229.0493 (-C_13_H_8_O_4_) by losing -H_2_O and -CO. As peaks 57, 59, 62, and 85 had the same fragments, they were presumed to be quercetin isomers.

The [M − H]^−^ ion of peak 66 at *m*/*z*: 599.1064 was in accordance with the molecular formula C_28_H_24_O_15_ in the negative ion mode. *m*/*z*: 599.1064 lost 152 Da to produce ion at *m*/*z*: 447.0969. The loss of 152 Da from *m*/*z*: 599.1064 and the fragment *m*/*z*: 169.0147 showed the presence of gallic acid units. Fragment *m*/*z*: 447.0969 lost 162 Da to form *m*/*z*: 285.0414, and fragment *m*/*z*: 313.0582 in MS/MS spectrum showed the presence of hexose units. Fragments *m*/*z*: 285.0414, 151.0036, and 125.0245 were consistent with quercetin cleavage. Therefore, peak 66 was identified as astragalin-*O*-gallate (Figure 2B1). As peak 73 had the same fragments, it was presumed to be an isomer of peak 66.

The [M − H]^−^ ion at *m*/*z*: 593.1341 of peak 89 was found in the negative ion MS/MS spectrum. Fragments *m*/*z*: 285.0399 and 151.0038 were consistent with kaempferol cleavage. Combined with *m*/*z*: 307.0835, 163.0398, and 145.0296, we speculated that this was tribuloside (C_30_H_26_O_13_) (Figure 2B2), and that peaks 84 and 93 were its isomers.

#### 3.1.3. Fragmentation of Quinine Acid Compounds

Nine caffeoylquinic acid compounds, which lost CO (*m*/*z* 28), CO_2 _(*m*/*z* 44), and H_2_O (*m*/*z* 18) during MS/MS cleavage, were observed temporarily in this study [30,31,32]. For example, [M + H]^+^ ion at *m*/*z*: 193.0706 of peak 3 was matched with the molecular formula, C_7_H_12_O_6_. Compared with [M + H]^+^ ions, *m*/*z*:175.0608 ions were less than 18 Da and *m*/*z*: 147.0656 ions were less than 46 Da, which meant that peak 3 contained hydroxyl and carboxyl groups. After comparison with standard products, peak 3 was identified as quinic acid.

The negative MS/MS spectra of peaks 22, 28, 37, and 68 showed the presence of quinic and caffeic acids in the structure. For example, in the MS/MS spectra of peak 22, the [M − H]^−^ ion at *m*/*z*: 353.0878 lost 162 Da and acquired *m*/*z* 191.0565, and lost 180 Da and acquired *m*/*z* 173.0445, which confirmed the presence of caffeic acid. In addition, [M − H]^−^ ion lost 192 Da and acquired *m*/*z* 161.0242, and lost 174 Da and acquired *m*/*z* 179.0344, which indicated the presence of quinic acid. Therefore, peaks 22, 28, and 37 were identified as caffeoylquinic acid isomers (C_16_H_18_O_9_) and peak 68 was dicaffeoylquinic acid (C_25_H_24_O_12_).

### 3.2. Structural Identification of Purified Samples

The 10% ethanol elution (101.2 g) was dissolved in water and then adsorbed by MCI-gel pore resin to obtain five fractions (Fr1–Fr5) by gradient elution of methanol/water (1:0–0:1). Fr1 underwent repeated ODS column chromatography and preparative HPLC to give compound **1**. Compound **2** was obtained by repeated crystallization of Fr3. Separation of Fr4 by ODS column chromatography gave compound **3**.

The 70% ethanol elution (516.2 g) was dissolved in methanol, subjected to column chromatography on silica gel (300–400 mesh) under reduced pressure, and eluted with ethyl acetate:methanol (1:0–1:1) to obtain 10 fractions (Fr1–Fr10). MCI columns, silica gel, ODS, Sephadex LH-20 and re-crystallization chromatographic methods were then use to separate compounds **3**–**8** by Fr1, compounds **9**–**12** by Fr2, compounds **13**–**16** by Fr3, and compounds **17** and **18** by Fr4.

A total of 18 purified compounds were identified using UV, MS, ^1^H-NMR, and ^13^C-NMR methods: 1,2,3,4,6-penta-*O*-galloyl-β-d-glucose (**1**), 3,4,5-trimethoxyphenyl-(6′-*O*-galloyl)-*O*-β-d-glucopyranoside (**2**), 6′-*O*-galloylsalidroside (**3**), gallic acid (**4**), protocatechuic acid (**5**), ethyl gallate (**6**), tiliroside (**7**), 3-*O*-coumaroylquinicacid (**8**), kaempferol-3-*O*-β-d-galactopyranosyl-(1→6)-β-d-glucopyranoside (**9**), kaempferol-3-*O*-β-d-galactopyranoside (**10**), quercetin-3-*O*-β-d-glucopyranoside (**11**), 5-*O*-caffeoylquinic acid (**12**), 3-*O*-coumaroylquinic acid methyl ester (**13**), 3-*O*-caffeoylquinic acid (**14**), 5-*O*-coumaroylquinic acid (**15**), 3,5-*O*-diocaffeoylquinic acid (**16**), 4,5-*O*-diocaffeoylquinic acid (**17**), 3,4-*O*-diocaffeoylquinic acid (**18**) (Supplementary Material Figure S1). Preliminary information on compounds **7**–**10**, **13,** and **15**–**18** has been reported elsewhere [33].

**Compound 1**: ESI-MS *m*/*z*: 939.1109 [M − H]^−^; ^1^H-NMR (600 MHz, DMSO) δ: 6.98 (2H, s), 6.92 (2H, s), 6.86 (2H, s), 6.82 (2H, s), 6.77 (2H, s), 6.38 (1H, d, *J* = 8.3 Hz, H-1), 5.95 (1H, t, *J* = 9.6 Hz, H-2), 5.43 (2H, m, H-3,4), 4.99 (1H, d, *J* = 9.8 Hz, H-5), 4.70 (2H, m, H_2_-6).^13^C-NMR (DMSO, 151 MHz) *δ*: 145.97 (C-1′ × 5), 139.58 (C-2′ × 5), 118.54 (C-3′,5′ × 5), 109.35 (C-2′,6′ × 5), 165.05 (C-7′ × 5), 92.16 (C-1), 72.39 (C-2), 72.59 (C-3), 68.23 (C-4), 71.03 (C-5) and 61.92 (C-6). By comparing experimental data with the literature [34], we determined compound **1** to be 1,2,3,4,6-penta-*O*-galloyl-β-d-glucose (C_41_H_32_O_26_).

**Compound 2**: ESI-MS *m*/*z*: 497.1300 [M − H]^−^. ^1^H-NMR (600 MHz, DMSO) δ: 6.30 (2H, s, H-2, 6), 3.64 (6H, s, OMe-3,5), 3.56 (3H, s, OMe-4), 4.92 (1H, d, *J *= 7.7 Hz, H-1′), 3.74 (1H, m, H-2′), 3.30 (3H, m, H-3′,4′,5′), 4.48 (1H, dd, *J* = 11.9 Hz, 1.7 Hz, H-6′a), 4.27 (1H, dd, *J* = 12.0, 5.7 Hz, H-6′b), 6.949 (2H, s, H-2″,6″), 9.25 (2H, br.s, OH-3″,5″), 8.95 (1H, br.s, OH-4″); ^13^C-NMR (151 MHz, DMSO) δ: 154.20 (C-1), 94.71 (C-2,6), 153.61 (C-3,5), 133.08 (C-4), 60.57 (3,5-OMe), 56.13 (4-OMe), 101.14 (C-1′), 73.66 (C-2′), 76.68 (C-3′), 70.17 (C-4′), 74.33 (C-5′), 64.04 (C-6′), 119.87 (C-1″), 109.08 (C-2″, 6″), 146.03 (C-3″, 5″), 138.95 (C-4″) and 166.27 (C-7″). Marrying the experimental data with the literature [35], compound **2** was identified as 3,4,5-trimethoxyphenyl-(6′-*O*-galloyl)-*O*-β-d-glucopyranoside (C_22_H_26_O_13_).

**Compound 3**: ESI-MS *m*/*z*: 451.1244 [M − H]^−^.^1 ^H-NMR (600 MHz, MeOD) δ: 6.66 (2H, d, *J* = 8.5 Hz, H-2,6), 6.98 (2H, d, *J* = 8.5 Hz, H-3,5), 3.71 (1H, m, Ha-7), 3.93 (1H, m, Hb-7), 2.80 (2H, m, H_2_-8), 4.33 (1H, d, *J* = 7.8 Hz, H-1′), 3.23 (1H, t, *J* = 8.4 Hz, H-2′), 3.41 (2H, m, H-3′,4′), 3.57 (1H, m, H-5′), 4.53 (1H, dd, *J* = 11.8, 2.1 Hz, Ha-6′), 4.44 (1H, dd, *J* = 11.8, 5.8 Hz, Hb-6′), 7.11 (2H, s, H-2″, 6″); ^13^C-NMR (151 MHz, MeOD) δ: 167.01 (COOH), 129.27 (C-1), 129.51 (C-2,6), 114.77 (C-3,5), 155.27 (C-4), 70.39 (C-7), 35.05 (C-8), 103.12 (C-1′), 73.70 (C-2′), 76.58 (C-3′), 70.88 (C-4′), 74.08 (C-5′), 63.40 (C-6′), 120.09 (C-1″), 108.84 (C-2″,6″), 145.15 (C-3″,5″) and 138.49 (C-4″). By comparing experimental data with the literature [36], compound **3** was identified as 6′-*O*-galloylsalidroside (C_21_H_24_O_11_).

**Compound 4**: ESI-MS *m*/*z*: 169.0145 [M − H]^−^.^1^H-NMR (600 MHz, DMSO) *δ*: 12.20 (1H, br.s, COOH), 9.15 (2H, br.s, OH-3,5), 8.80 (1H, br.s, OH-4), 6.937 ( 2H, s, H-2,6); ^13^C-NMR (DMSO,151 MHz)* δ*: 120.90 (C-1),109.19 (C-2,6), 145.87 (C-3,5), 138.45 (C-4), 170.6 (C-7). Merging experimental and literature data [37], revealed compound **4** to be gallic acid (C_7_H_6_O_5_).

**Compound 5**: ESI-MS *m*/*z*:153.0197 [M − H]^−^. ^1^H-NMR (600 MHz, MeOD) δ: 12.20 (1H, br.s, COOH), 8.48 (2H, br.s, OH-3,4), 7.47 (1H, d, *J* = 2.0 Hz, H-2), 6.82 (1H, d, *J* = 8.0 Hz, H-5), 7.45 (1H, dd, *J* = 8.2, 2.1 Hz, H-6);^ 13^C-NMR (151 MHz, MeOD) δ: 122.44 (C-1), 108.95 (C-2), 149.92 (C-3), 144.60 (C-4), 114.34 (C-5), 116.37 (C-6) and 169.33 (C-7). According to the combined experimental and literature data [38], it was determined that compound **5** was protocatechuic acid (C_7_H_6_O_4_).

**Compound 6**: ESI-MS *m*/*z*: 197.0453 [M − H]^−^. ^1^H-NMR (600 MHz, DMSO) δ: 6.97 (2H, s, H-2,6), 4.22 (2H, q, *J* = 7.1 Hz, CH_2_), 1.28 (3H, t, *J* = 7.1 Hz, CH_3_); ^13^C-NMR (151 MHz, DMSO) δ: 120.80 (C-1), 108.96 (C-2,6), 146.03 (C-3,5), 138.82 (C-4), 166.30 (C-7), 60.46 (CH_2_) and 14.72 (CH_3_). Based on the above data and the literature [39], compound **6** was identified as ethyl gallate (C_9_H_10_O_5_).

**Compound 11**: ESI-MS *m*/*z*: 463.0882 [M − H]^−^. ^1^H-NMR (600 MHz, DMSO) δ: 7.70 (1H, d, *J* = 2.4 Hz, H-2′), 7.56 (1H, dd, *J* = 2.4, 8.8 Hz, H-6′), 6.84 (1H, d, *J* = 8.6 Hz, H-5′), 6.40 (1H, d, *J* = 2.0 Hz, H-8), 6.20 (1H, d, *J* = 2.0 Hz, H-6), 5.45 (1H, d, *J* = 7.4 Hz, H-1″), 3.22–3.59 (6H, m, H-2″−6″); ^13^C-NMR (151 MHz, DMSO) δ: 156.81 (C-2), 133.80 (C-3), 177.91 (C-4), 161.72 (C-5), 99.15 (C-6), 164.68 (C-7), 93.98 (C-8), 156.64 (C-9), 104.43 (C-10), 122.07 (C-1′), 115.68 (C-2′), 121.65 (C-6′), 116.68 (C-5′), 148.94 (C-4′), 101.36 (C-1″), 74.58 (C-2″), 76.99 (C-3″), 70.42 (C-4″), 78.04 (C-5″) and 61.46 (C-6″). Based on our findings and the literature [40], compound **11** was identified as quercetin-3-*O*-β-d-glucopyranoside (C_21_H_20_O_12_).

**Compound 12**: ESI-MS *m*/*z* 353.0879 [M − H]^−^. ^1^H-NMR (600 MHz, MeOD) δ: 2.24 (2H, m, H-2), 4.19 (1H, br.s, H-3), 3.76 (1H, m, H-4), 5.37 (1H, d, *J* = 4.2 Hz, H-5), 2.10 (2H, m, H-6), 7. 06 (1H, d, *J* = 1.9 Hz, H-2′), 6.79 (1H, d, *J* = 8.2 Hz, H-5′), 6.98 (1H, dd, *J* = 1.9, 8.2 Hz, H-6′), 7.58 (1H, d, *J* = 15.9 Hz, H-7′), 6.28 (1H, d, *J* = 15.9 Hz, H-8′); ^13^C-NMR (151 MHz, DMSO) δ: 74.84 (C-1), 37.47 (C-2), 69.98 (C-3), 70.61 (C-4), 72.15 (C-5), 36.84 (C-6), 175.73 (C-7), 126.43 (C-1′), 113.90 (C-2′), 145.41 (C-3′), 148.17 (C-4′), 115.10 (C-5′), 121.58 (C-6′), 145.69 (C-7′), 113.83 (C-8′) and 167.30 (C-9′). Based on the above data and the literature [41], compound **12** was identified as 5-*O*-caffeoylquinic acid (C_16_H_18_O_9_).

**Compound 14**: ESI-MS *m*/*z* 353.0881 [M − H]^−^. ^1^H-NMR (600 MHz, DMSO) δ: 1.79 (2H, m, H-2), 5.07 (1H, m, H-3), 3.78 (1H, m, H-4), 4.75 (1H, br.s, H-5), 1.95 (2H, m, H-6), 7.04 (1H, d, *J* = 1. 9 Hz, H-2′), 6.77 (1H, d, *J* = 8.1 Hz, H-5′), 6.98 (1H, dd, *J* = 1.9, 8.2 Hz, H-6′), 7.42 (1H, d, *J* = 15.9 Hz, H-7′), 6.15 (1H, d, *J* = 15.9 Hz, H-8′); ^13^C-NMR (151 MHz, DMSO) δ: 73.95 (C-1), 37.68 (C-2), 71.34 (C-3), 70.85 (C-4), 68.54 (C-5), 36.72 (C-6), 175.38 (C-7), 126.07 (C-1′), 115.25 (C-2′), 145.40 (C-3′), 148.81 (C-4′), 116.21 (C-5′), 121.81 (C-6′), 146.03 (C-7′), 114.77 (C-8′) and 166.19 (C-9′). Based on the above data and literature findings [41], compound **14** was identified as 3-*O*-caffeoylquinic acid (C_16_H_18_O_9_).

### 3.3. Antioxidant Activity Analysis of Purified Compounds

**Figure 3:**
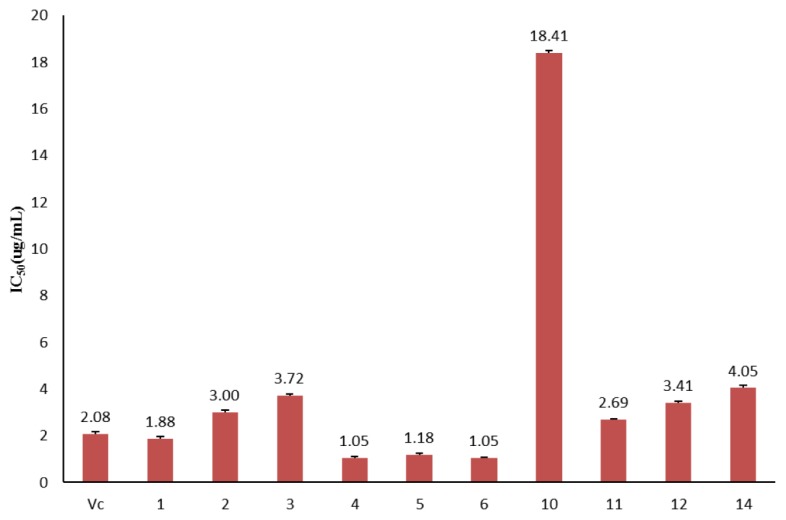
The IC_50_ values of purified compounds in LCO on antioxidant activity.

The antioxidant activity of the purified compounds was tested using DPPH methods (Table 2, Figure 3). With the exception of compound **11**, all test compounds showed significant antioxidant activity. The IC_50_ values of compounds **2**, **3**, **11**, **12,** and **14** ranged from 3.00 to 4.05 μg/mL, and all were slightly higher than in the V_C_ control group (2.08 μg/mL). The IC_50_ values of compounds **1**, **4**, **5,** and **6** were 1.88, 1.05, 1.18, and 1.05 μg/mL, respectively, and were significantly lower than those of the V_C_ control group.

## 4. Conclusions

In this study, UHPLC-Q-TOF-MS/MS and a variety of chromatographic separation techniques were used to systematically analyze and separate the active antioxidant components of LCO. A total of 100 compounds were identified from the 70% ethanol extract of LCO, and these were mainly gallic acid tannins and flavonoids. Of these, 18 pure compounds were isolated, with compounds **2**, **5**, **6**, **12**, **14,** and **16**–**18** identified for the first time in LCO and the genus Loropetalum. DPPH results showed that compounds **1**, **4**, **5,** and **6** had significant antioxidant activity.

## Figures and Tables

**Table 1:** Identification of the chemical constituents in *Loropetalum chinense *(*R. Brown*) Oliv. (LCO) by HPLC–ESI-Q-TOF-MS/MS.

No.	tR/Min	Formula	Error/ppm	Adduct	Found at Mass/Da	MS^2^ Ions	Indentification
1	1.21	C_14_H_16_O_10_	−0.1	[M + H]^+^	345.0815	171.0275, 153.0179, 125.0235	Theogallin
2	1.32	C_13_H_16_O_10_	0.2	[M − H]^−^	331.0671	331.0671, 169.0144, 125.0243	Monogalloyl glucose
3	1.32	C_7_H_12_O_6_	−0.8	[M + H]^+^	193.0706	175.0608, 147.0656, 129.0551	Quinic acid
4	1.66	C_14_H_16_O_10_	0.5	[M + H]^+^	345.0818	327.0726, 171.0289, 153.0185, 125.0232	Theogallin
5	1.75	C_7_H_6_O_5_	−2.4	[M + H]^+^	171.0284	153.0180, 135.0074, 125.0234, 109.0286, 107.0128, 97.0283	Gallic acid
6	1.97	C_13_H_16_O_10_	0.7	[M − H]^−^	331.0673	331.0673, 169.0130, 125.0241	Monogalloyl glucose
7	2.35	C_13_H_16_O_10_	−0.1	[M − H]^−^	331.0672	331.0672, 169.0144, 125.0242	Monogalloyl glucose
8	2.86	C_13_H_16_O_10_	−0.1	[M − H]^−^	331.0671	331.0671, 169.0142, 125.0249	Monogalloyl glucose
9	3.63	C_7_H_6_O_4_	−1.7	[M + H]^+^	155.0336	137.0230, 109.0288, 107.0112, 93.0343, 81.0343	Protocatechuic acid
10	3.63	C_27_H_22_O_18_	2.6	[M − H]^−^	633.0750	633.0750, 481.0649, 300.0992, 169.0153, 125.0232	Corilagin
11	3.64	C_13_H_16_O_10_	0.9	[M − H]^−^	331.0674	331.0685, 169.0148, 151.0031, 125.0257	Monogalloyl glucose
12	4.06	C_20_H_20_O_14_	1	[M − H]^−^	483.0785	483.0786, 331.0673, 313.0568, 169.0145, 151.0038, 125.0249	Digalloylglucose
13	4.07	C_19_H_26_O_15_	0.3	[M − H]^−^	493.1201	493.1201, 331.0648, 169.0142, 125.0248	Gallic acid diglucoside
14	4.26	C_13_H_16_O_10_	−0.1	[M − H]^−^	331.0670	331.0670, 169.0141, 125.0250	Monogalloyl glucose
15	4.49	C_19_H_26_O_15_	1	[M − H]^−^	493.1204	493.1204, 331.0688, 313.0565, 169.0149, 125.0243	Gallic acid diglucoside
16	4.52	C_27_H_22_O_18_	3.4	[M − H]^−^	633.0752	633.0752, 481.0608, 463.0505, 300.0992, 169.0138, 125.0255	Corilagin
17	4.77	C_8_H_8_O_5_	−3.8	[M + H]^+^	185.0437	125.0305, 107.0121, 81.0340	Methyl gallate
18	4.79	C_20_H_20_O_14_	0.8	[M − H]^−^	483.0784	483.0784, 331.0676, 313.0566, 169.0150, 151.0041, 125.0254	Digalloylglucose
19	5.31	C_27_H_22_O_18_	3.5	[M − H]^−^	633.0755	633.0755, 463.0597, 300.0980, 169.0152	Corilagin
20	5.33	C_20_H_20_O_14_	1.2	[M − H]^−^	483.0786	483.0786, 331.0677, 313.0571, 169.0143, 151.0035, 125.0249	Digalloylglucose
21	5.81	C_27_H_22_O_18_	2.8	[M − H]^−^	633.0751	633.0751, 481.0757, 463.0505, 300.0989, 169.0143, 125.0246	Corilagin
22	5.89	C_16_H_18_O_9_	0.7	[M − H]^−^	353.0889	191.0565, 179.0347, 173.0445, 161.0249, 135.0447	Caffeoylquinic acid
23	5.99	C_27_H_24_O_18_	−3	[M − H]^−^	635.0955	635.0955, 483.0799, 465.0670, 331.0694, 313.0574, 169.0143, 125.0253	Trigalloylglucopyranose
24	6.02	C_14_H_10_O_9_	−0.8	[M + H]^+^	323.0395	153.0179, 125.0233, 79.0185	Digallic acid
25	6.84	C_8_H_8_O_5_	−1.1	[M + H]^+^	185.0442	153.0183, 135.0078, 125.0236, 107.0103, 97.0282	Methyl gallate
26	6.88	C_27_H_24_O_18_	2.9	[M − H]^−^	635.0908	635.0908, 483.0788, 465.0700, 331.0683, 313.0579, 169.0149, 125.0252	Trigalloylglucopyranose
27	6.92	C_27_H_22_O_18_	3.5	[M − H]^−^	633.0749	633.0749, 300.0981, 169.0150, 125.0239	Corilagin
28	7.09	C_16_H_18_O_9_	0.3	[M − H]^−^	353.0878	191.0554, 179.0344, 173.0446, 161.0242, 135.0447	Chlorogenic acid
29	7.43	C_27_H_30_O_16_	−1.1	[M + H]^+^	611.1784	611.1784, 449.1066, 287.0559	Panasenoside
30	7.50	C_27_H_24_O_18_	3.3	[M − H]^−^	635.0910	635.0913, 483.0789, 465.0687, 331.0698, 313.0567, 169.0145, 125.0241	Trigalloylglucopyranose
31	7.51	C_27_H_22_O_18_	3.2	[M − H]^−^	633.0753	633.0753, 481.0729, 300.0991, 169.0145, 125.0255	Corilagin
32	7.63	C_9_H_10_O_5_	−2	[M + H]^+^	199.0597	181.0502, 153.0190, 140.0470, 125.0232, 107.0121, 97.0288	Ethyl gallate
33	7.87	C_15_H_10_O_5_	0.2	[M + H]^+^	271.0599	271.0599, 215.0699, 177.0559, 169.0641, 153.0569, 149.0246, 119.0509	Apigenin
34	7.87	C_22_H_18_O_11_	−1.4	[M + H]^+^	459.0915	459.0915, 307.0381, 289.0714, 163.0391, 153.0178, 151.0391, 139.0389	Epigallocatechin Gallate
35	7.97	C_27_H_30_O_16_	−2.2	[M + H]^+^	611.1593	449.1059, 303.0484, 287.0534, 267.0019, 145.0489, 85.0278	Rutin
36	8.14	C_16_H_18_O_8_	0.3	[M + H]^+^	339.1073	165.0545, 147.0441, 119.0489, 91.0548, 65.0396	Coumaroylquinic acid
37	8.18	C_16_H_18_O_9_	0.4	[M − H]^−^	353.0878	191.0558, 179.0328, 173.0328, 161.0243, 135.0456	Caffeoylquinic acid
38	8.18	C_27_H_24_O_18_	3.3	[M − H]^−^	635.0911	635.0911, 483.0789, 465.0690, 313.0567, 169.0143, 125.0247	Trigalloylglucopyranose
39	8.97	C_21_H_24_O_11_	−1.1	[M + H]^+^	453.1386	453.1386, 406.9998, 315.0727, 297.0602, 255.0507, 171.0283, 153.0178, 127.0393	Galloylsalidroside
40	9.08	C_17_H_20_O_9_	−0.8	[M + H]^+^	369.1177	195.0656, 177.0547, 149.0588, 145.0283, 134.0349, 117.0344, 89.0394	Feruloylquinic acid
41	9.10	C_27_H_24_O_18_	1.7	[M − H]^−^	635.0901	635.0901, 483.0800, 465.0679, 331.0694, 313.0566, 169.0140, 125.0253	Trigalloylglucopyranose
42	9.20	C_34_H_28_O_22_	2.8	[M − H]^−^	787.1021	787.1021, 635.0919, 617.0798, 483.0752, 465.0682, 313.0566, 169.0142, 125.0249	Tetrakisgalloylglucopyranose
43	9.35	C_16_H_18_O_8_	0.3	[M + H]^+^	339.1073	147.0440, 119.0492, 91.0548, 65.0395	Coumaroylquinic acid
44	9.85	C_41_H_32_O_26_	−6.3	[M − H]^−^	939.1050	939.1050, 787.0948, 769.0879, 635.0942, 617.0738, 313.0576, 169.0147	Pentagalloylglucopyranose
45	10.00	C_34_H_28_O_22_	3.4	[M − H]^−^	787.1026	787.1026, 635.0921, 617.0811, 483.0785, 465.0679, 313.0555, 169.0141, 125.0245	Tetrakisgalloylglucopyranose
46	10.21	C_22_H_26_O_13_	−0.9	[M + H]^+^	499.1442	315.0719, 297.0616, 275.0925, 255.0505, 185.0814, 171.0294, 153.0189, 127.0389	1-*O*-3′,4′,5′-trimethoxyphenyl-(6-*O*-galloyl)-β-d-glucopyranoside
47	10.22	C_28_H_24_O_16_	−0.9	[M + H]^+^	617.1131	617.1131, 447.0910, 303.0503, 297.0610, 153.0184	Galloylhyperin
48	10.29	C_34_H_28_O_22_	3.5	[M − H]^−^	787.1027	787.1027, 635.0934, 617.0827, 483.0827, 465.0697, 313.0571, 169.0152, 125.0255	Tetrakisgalloylglucopyranose
49	10.51	C_28_H_24_O_16_	−1.9	[M + H]^+^	617.1129	617.1147, 447.0920, 237.0385, 153.0189	Galloylhyperin
50	10.51	C_22_H_18_O_10_	−0.9	[M + H]^+^	443.0969	273.0757, 165.0549, 153.0179, 151.0386, 147.0431, 139.0385, 123.0438	Catechin gallate
51	10.52	C_15_H_12_O_5_	−0.3	[M + H]^+^	273.0757	273.0760, 163.0402, 153.0192, 147.0424, 135.0436, 123.0440, 105.0326	Naringenin
52	10.63	C_21_H_18_O_13_	−1.2	[M + H]^+^	479.0814	317.0294, 285.0024, 257.0061	Shikimic acid-*O*-digallate
53	10.63	C_34_H_28_O_22_	3.6	[M − H]^−^	787.1028	787.1028, 635.0925, 617.0823, 483.0829, 465.0656, 313.0557, 169.0143, 125.0246	Tetrakisgalloylglucopyranose
54	10.76	C_14_H_6_O_8_	0.3	[M + H]^+^	303.0140	303.0140, 285.0035, 275.0189, 257.0082, 229.0130, 201.0179, 173.0232, 145.0284	Ellagic acid
55	10.78	C_15_H_10_O_8_	0.1	[M + H]^+^	319.0449	319.0450, 301.0346, 290.0422, 273.0393, 245.0444, 217.0503, 165.0176, 153.0182	Myricetin
56	10.94	C_21_H_20_O_12_	−0.6	[M + H]^+^	465.1028	319.0453, 303.0507, 285.0388, 257.0455, 229.0497, 145.0498, 127.0387, 97.0290	Myricitrin
57	10.96	C_15_H_10_O_7_	−0.7	[M + H]^+^	303.0497	303.0497, 285.0037, 275.0176, 257.0436, 229.0493, 153.0181 137.0229	Isomer of Quercetin
58	11.21	C_21_H_20_O_12_	−0.7	[M + H]^+^	465.1025	303.0508, 257.0446, 229.0492, 165.0176, 145.0491, 127.0389, 97.0289, 85.0289	Hyperoside
59	11.22	C_15_H_10_O_7_	0.2	[M + H]^+^	303.0503	303.0503, 285.0389, 257.0457, 229.0498, 153.0181 137.0232	Isomer of Quercetin
60	11.40	C_21_H_20_O_11_	−1.1	[M + H]^+^	449.1073	287.0545, 153.0178	Luteoloside
61	11.66	C_41_H_32_O_26_	2.4	[M − H]^−^	939.1131	939.1131, 787.0943, 769.0877, 635.0796, 617.0789, 313.0644, 169.0141, 125.0259	Pentagalloylglucopyranose
62	11.73	C_15_H_10_O_7_	−0.7	[M + H]^+^	303.0497	303.0497, 285.0367, 257.0453, 229.0478, 153.0186, 137.0591	Isomer of Quercetin
63	11.96	C_20_H_18_O_11_	−1	[M + H]^+^	435.092	303.0500, 285.0415, 257.0448, 229.0493, 153.0179, 137.0217	Isomer of guaijaverin
64	12.06	C_15_H_10_O_6_	0.2	[M + H]^+^	287.0542	287.0542, 258.0511, 241.0482, 213.0550, 165.0167, 153.0178, 137.0218, 121.0282	Isomer of Kaempferol
65	12.07	C_21_H_20_O_11_	−0.3	[M + H]^+^	449.1075	287.0560, 165.0177, 153.0182	Isomer of luteoloside
66	12.16	C_28_H_24_O_15_	6.6	[M − H]^−^	599.1088	599.1088, 447.0969, 313.0582, 285.0414, 169.0147, 151.0036, 125.0245	Astragalin-*O*-gallate
67	12.22	C_41_H_32_O_26_	2.6	[M − H]^−^	939.1133	939.1133, 787.1020, 769.0979, 635.0901, 617.0780, 313.0514, 169.0142, 125.0262	Pentagalloylglucopyranose
68	12.54	C_25_H_24_O_12_	0.6	[M − H]^−^	515.1195	515.1195, 353.0883, 191.0556, 179.0343, 135.0449	Dicaffeoylquinic acids
69	12.55	C_15_H_10_O_6_	0.3	[M + H]^+^	287.0552	287.0552, 258.0525, 241.0483, 213.0533, 165.0188, 157.0469, 153.0183, 121.0285	Isomer of Kaempferol
70	12.63	C_21_H_20_O_11_	−0.7	[M + H]^+^	449.1075	303.0511, 287.0562, 165.0178, 145.0496, 129.0548, 127.0387	Quercitrin
71	12.67	C_15_H_10_O_7_	0.6	[M + H]^+^	303.0505	303.0505, 285.0395, 257.0445, 229.0494, 153.0182, 137.0231	Isomer of Quercetin
72	12.97	C_16_H_12_O_7_	1.8	[M + H]^+^	317.0653	317.0653, 302.0437, 285.0415, 274.0482, 246.0531, 229.0483, 153.0182	Isomer of isorhamnetin
73	12.99	C_28_H_24_O_15_	7.6	[M − H]^−^	599.1064	599.1064, 447.0947, 313.0577, 285.0408, 169.0136, 151.0035, 125.0238	Astragalin-*O*-gallate
74	13.02	C_15_H_10_O_6_	0.4	[M + H]^+^	287.0552	287.0552, 258.0533, 231.0647, 213.0545, 165.0171, 153.0175, 137.0236, 121.0286	Isomer of Kaempferol
75	13.04	C_35_H_28_O_19_	−1.8	[M + H]^+^	753.1284	467.0822.449.0706, 315.0705, 287.0552, 153.0181, 125.0236	Astragalin-*O*-digallate
76	13.30	C_15_H_10_O_6_	0.4	[M + H]^+^	287.0561	287.0561, 258.0566, 213.0567, 165.0193, 153.0175, 147.0429, 137.0240	Isomer of Kaempferol
77	13.68	C_23_H_24_O_12_	−1.6	[M + H]^+^	493.1332	493.1332, 331.0819, 315.0505, 270.0515	Tricin-*O*-glucopyranoside
78	13.72	C_35_H_28_O_19_	−2	[M + H]^+^	753.1283	753.1261, 601.1206, 467.0812, 449.0707, 287.0547, 237.0393, 153.0185	Astragalin-*O*-digallate
79	14.27	C_15_H_10_O_6_	0.5	[M + H]^+^	287.0546	287.0546, 258.0527, 241.0491, 213.0551, 165.0180, 153.0181, 137.0233, 121.0285	Isomer of Kaempferol
80	14.27	C_21_H_20_O_10_	−1	[M + H]^+^	433.1125	287.0553, 165.0183, 129.0542, 85.0285, 71.0498	Kaempferol-*O*-rhamnoside
81	14.32	C_23_H_24_O_12_	−1.5	[M + H]^+^	493.3333	331.0814, 315.0496, 270.0519	Isomer of tricin-*O*-glucopyranoside
82	14.32	C_35_H_28_O_19_	−2.5	[M + H]^+^	753.1279	753.1366, 601.1066, 467.0804, 449.0691, 287.0543, 237.0403, 153.0183	Isomer of astragalin-di-*O*-gallate
83	14.64	C_16_H_12_O_7_	1.3	[M + H]^+^	317.0649	317.0649, 302.0442, 285.0392, 246.0505, 175.9679, 153.0188, 139.0399	Isomer of isorhamnetin
84	14.69	C_30_H_26_O_13_	9.4	[M − H]^−^	593.1360	593.1360, 447.0949, 307.0825, 285.0413, 163.0403, 151.0030, 145.0290, 119.0508	Isomer of Tribuloside
85	16.27	C_15_H_10_O_7_	0.4	[M + H]^+^	303.0503	303.0503, 285.0399, 257.0445, 229.0491, 201.0552, 153.0182, 137.0230	Quercetin
86	16.36	C_15_H_10_O_6_	−0.6	[M + H]^+^	287.0553	287.0553, 161.0234, 153.0183, 135.0434	Isomer of Kaempferol
87	16.72	C_16_H_12_O_7_	−0.7	[M + H]^+^	317.0569	317.0569, 302.0420, 274.0469, 228.0420, 153.0170, 147.0435	Isorhamnetin
88	16.88	C_15_H_10_O_6_	0.8	[M + H]^+^	287.0547	287.0547, 258.0507, 241.0461, 213.0539, 165.0182, 153.0179, 121.0281	Isomer of Kaempferol
89	16.89	C_30_H_26_O_13_	6.8	[M − H]^−^	593.1341	593.1341, 447.0955, 307.0835, 285.0399, 163.0398, 151.0038, 145.0296, 119.0506	Tribuloside
90	16.89	C_22_H_22_O_10_	−1.3	[M + H]^+^	447.128	301.0705, 286.0479, 258.0536, 153.0179	Methylluteolin-*O*-rhamnopyranosid
91	17.34	C_17_H_14_O_7_	−0.2	[M + H]^+^	331.0812	331.0820, 315.0498, 286.0462, 270.0522, 258.0520	Quercetin-dimethyl ether
92	17.39	C_15_H_10_O_6_	0.8	[M + H]^+^	287.055	287.0550, 258.0539, 241.0463, 213.0522, 165.0175, 153.0190, 121.0279	Isomer of Kaempferol
93	17.39	C_30_H_26_O_13_	5.8	[M − H]^−^	593.1354	593.1354, 447.0938, 307.0828, 285.0403, 163.0396, 151.0031, 145.0290, 119.0505	Isomer of Tribuloside
94	17.48	C_22_H_22_O_10_	−0.9	[M + H]^+^	447.1281	301.0710, 286.0490, 258.0527	Methylluteolin-*O*-rhamnopyranosid
95	18.11	C_15_H_12_O_5_	−0.8	[M + H]^+^	273.0755	273.0736, 164.8737, 153.0183, 147.0432, 121.0273, 91.0557	Isomer of naringenin
96	19.05	C_15_H_10_O_6_	0.9	[M + H]^+^	287.0557	287.0557, 258.0527, 241.0494, 231.0652, 213.0550, 165.0185, 153.0184, 121.0286	Kaempferol
97	19.52	C_17_H_14_O_7_	0.7	[M + H]^+^	331.0183	331.0819, 315.0505, 286.0477, 270.0529, 258.0529, 242.0583	Quercetin-dimethyl ether
98	19.76	C_16_H_12_O_7_	−0.9	[M + H]^+^	317.0657	317.0657, 302.0424, 274.0464, 153.0185	Isomer of isorhamnetin
99	19.97	C_20_H_18_O_11_	−1.1	[M + H]^+^	435.0924	435.0924, 237.0398, 153.0175, 127.0406	Guaijaverin
100	20.71	C_17_H_14_O_7_	−0.3	[M + H]^+^	331.0809	331.0807, 315.0495, 286.0538, 270.0528, 242.0549, 168.0608	Quercetin-dimethyl ether

**Table 2:** The antioxidant activity of purified compounds.

Vc	Concentration (µg/mL)	0.73	1.16	1.45	2.18	2.90
	Inhibition rate (%)	18.71 ± 0.17	24.26 ± 0.36	34.79 ± 0.47	46.19 ± 0.28	69.15 ± 0.45
1	Concentration (µg/mL)	0.88	1.75	2.63	2.98	3.50
	Inhibition rate (%)	20.03 ± 0.15	37.09 ± 0.22	63.64 ± 0.26	69.15 ± 0.40	85.93 ± 0.11
2	Concentration (µg/mL)	2.31	2.78	3.24	3.70	4.63
	Inhibition rate (%)	29.08 ± 0.20	45.43 ± 0.31	52.63 ± 0.11	62.32 ± 0.35	87.81 ± 0.13
3	Concentration (µg/mL)	2.33	3.72	4.65	5.58	6.98
	Inhibition rate (%)	25.99 ± 0.14	46.53 ± 0.15	59.59 ± 0.31	71.43 ± 0.24	87.76 ± 0.16
4	Concentration (µg/mL)	0.52	0.87	1.21	1.56	2.08
	Inhibition rate (%)	22.33 ± 0.25	38.75 ± 0.27	52.23 ± 0.36	67.66 ± 0.40	82.14 ± 0.43
5	Concentration (µg/mL)	0.76	1.01	1.68	1.89	2.10
	Inhibition rate (%)	26.66 ± 0.37	38.02 ± 0.33	67.52 ± 0.28	78.21 ± 0.19	81.00 ± 0.40
6	Concentration (µg/mL)	0.61	1.01	1.41	1.82	2.02
	Inhibition rate (%)	26.25 ± 0.27	42.90 ± 0.32	59.54 ± 0.33	76.50 ± 0.27	86.47 ± 0.33
10	Concentration (µg/mL)	10.63	21.25	25.5	31.88	42.50
	Inhibition rate (%)	27.55 ± 0.29	50.94 ± 0.19	61.29 ± 0.26	78.36 ± 0.41	87.37 ± 0.37
11	Concentration (µg/mL)	1.38	3.22	3.68	4.14	9.20
	Inhibition rate (%)	25.40 ± 0.27	47.85 ± 0.26	63.16 ± 0.29	75.07 ± 0.33	88.54 ± 0.33
12	Concentration (µg/mL)	0.99	2.46	7.39	9.85	12.31
	Inhibition rate (%)	22.33 ± 0.39	46.55 ± 0.40	68.06 ± 0.50	72.53 ± 0.31	74.79 ± 0.25
14	Concentration (µg/mL)	2.23	3.56	4.45	6.68	8.01
	Inhibition rate (%)	27.06 ± 0.19	39.51 ± 0.51	46.28 ± 0.37	66.85 ± 0.24	88.77 ± 0.31

## Supplementary Materials

The following are available online. Figure S1: The structure of compound **1**~**18**.
