# Supplementary material for: Isolation and Identification of the Anti-Oxidant Constituents from Loropetalum chinense (R. Brown) Oliv. Based on UHPLC–Q-TOF-MS/MS
Source: Molecules. 2018 Jul 14;23(7):1720. doi: 10.3390/molecules23071720 (PMC6099825; doi:10.3390/molecules23071720)
Supplement: Supplementary file 1 [file molecules-23-01720-s001.pdf]

Isolation and identification of the anti-oxidant constituents from *Loropetalum chinense (R. Brown) Oliv.* based on UHPLC-Q-TOF-MS/MS

Haifang Chen [#], Mulan Li[#], Chen Zhang[#], Wendi Du, Haihua Shao, Yulin Feng[*], Wugang Zhang[*], Shilin Yang

Jiangxi University of Traditional Chinese Medicine, Nanchang 330006, China

[#] equally contributed to this article

* Correspondence: fengyulin2003@hotmail.com(Y.F.); Tel.: +86 079187119632(Y.F.); zwgchf98@foxmail.com (W.Z.); Tel.: +86 079187119638 (W.Z.)

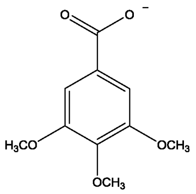

a

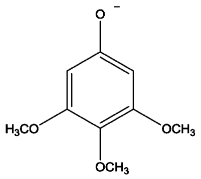

b

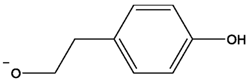

c

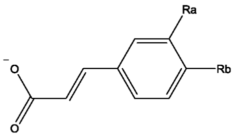

d

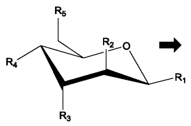

	R1	R2	R3	R4	R5
**1**	a	a	a	a	a
**2**	b	OH	OH	OH	a
**3**	c	OH	OH	OH	a

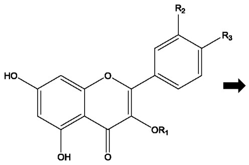

	R1	R2	R3	Ra	Rb
**7**	glc-6-O-d	H	OH	H	OH
**9**	glc-6-O-β-D-gal	H	OH	—	—
**10**	glc	H	OH	—	—
**11**	gal	OH	OH	—	—

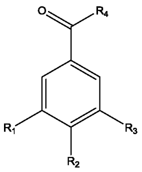

	R1	R2	R3	R4
**4**	OH	OH	OH	OH
**5**	H	OH	OH	OH
**6**	OH	OH	OH	OC_2H_5

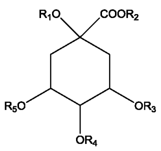

	R1	R2	R3	R4	R5	Ra	Rb
**8**	H	H	d	H	H	H	OH
**12**	H	H	H	H	d	OH	OH
**13**	H	CH_3	d	H	H	OH	OH
**14**	H	H	d	H	H	OH	OH
**15**	H	H	H	H	d	H	OH
**16**	H	H	d	H	d	OH	OH
**17**	H	H	H	d	d	OH	OH
**18**	H	H	d	d	H	OH	OH

Figure S1. The structure of compound 1~18.
